# Supplementary material for: FGF diffusion is required for directed migration of postembryonic muscle progenitors in C. elegans
Source: Development. 2025 Sep 22;152(18):dev204802. doi: 10.1242/dev.204802 (PMC12516322; doi:10.1242/dev.204802)
Supplement: Supplementary information [file develop-152-204802-s1.pdf]

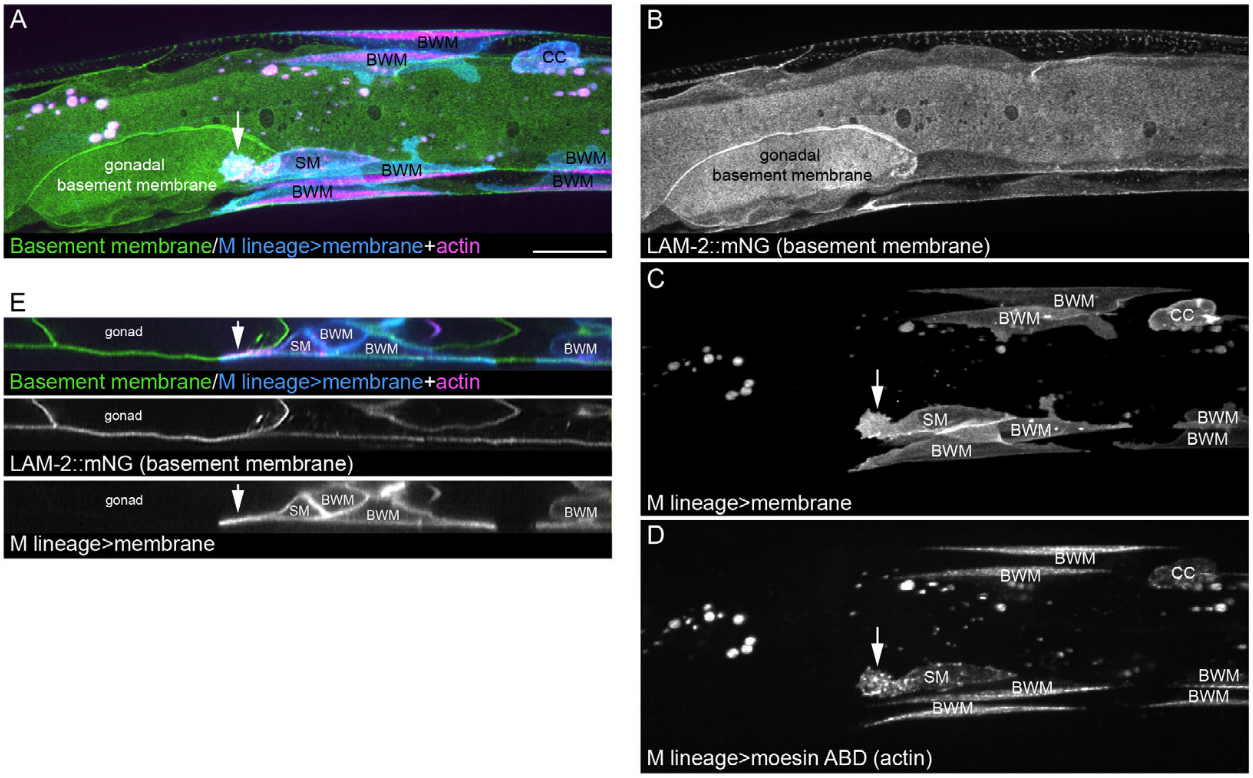

Fig. S1. The gonadal basement membrane separates migrating SMs from somatic gonad cells.

(A) Maximum intensity projection showing basement membranes outlined by LAM-2::mNG along with membranes and actin in M lineage cells. An SM protrusion (white arrow) has contacted the gonadal basement membrane. **(B-D)** Individual fluorescence channels showing LAM-2::mNG **(B)**, M lineage cell membranes outlined with 2x mTurquoise::PH **(C)**, and actin visualized with 2x mKate2::moesin ABD (D) in a *Phlh-8>2x mKate2::moesin ABD::F2A:: 2x mTurquoise2::PH x lam-2::mNG* animal. **(E)** Orthogonal slice projection of the same Z-stack showing an SM protrusion extending over the surface of the gonadal basement membrane as the SM crawls forwards. Animals are oriented with anterior to left and dorsal to top in A-D. E shows a coronal section with anterior to the left and the ventral midline to the top. Scale bar = 10 mm.

Development • Supplementary information

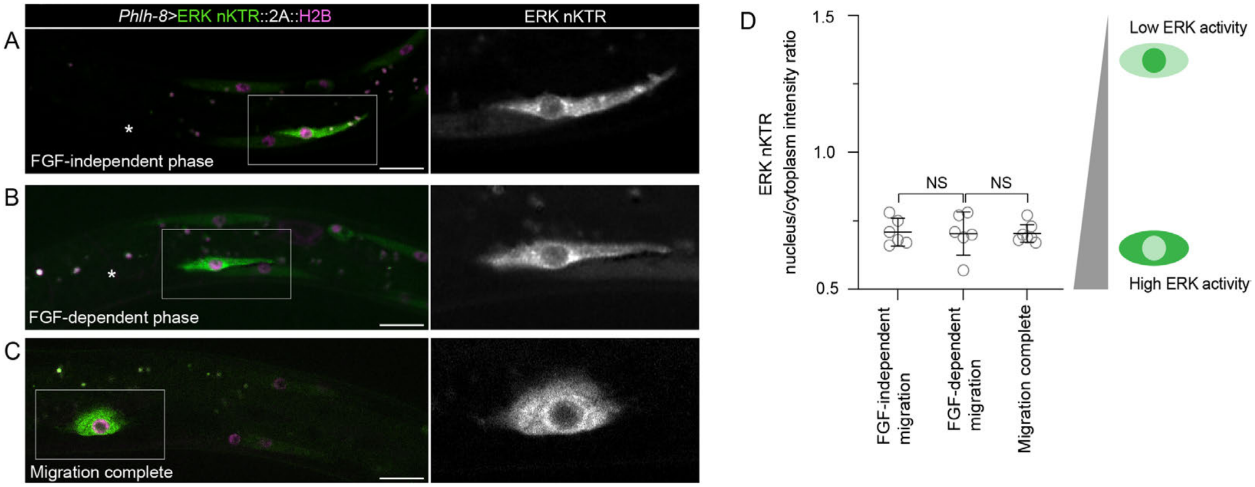

Fig. S2. ERK nKTR biosensor activity during SM migration.
Nuclear levels of ERK-nKTR provide a real-time, quantitative readout of ERK activity. **(A-C)** Live imaging of ERK-nKTR biosensor during the FGF independent **(A)** and FGF-dependent **(B)** stages of migration along with the migration endpoint **(C)**. **(D)** Quantification of ERK-nKTR nucleus/cytoplasm intensity ratio in SMs during FGF-independent early migration, FGF-dependent later migration, and after centering over the somatic gonad. Y-axis is scaled to the highest and lowest values observed across M lineage cell types, and lower nucleus/cytoplasm ratios indicate higher levels of ERK activity. NS = Not significant (Kolmogorov Smirnov test). P=0.9307 for FGF-independent vs. FGF-dependent migration timepoints and P=0.8741 for FGF-dependent migration vs migration endpoint. Scale bars = 10 mm.

Development • Supplementary information

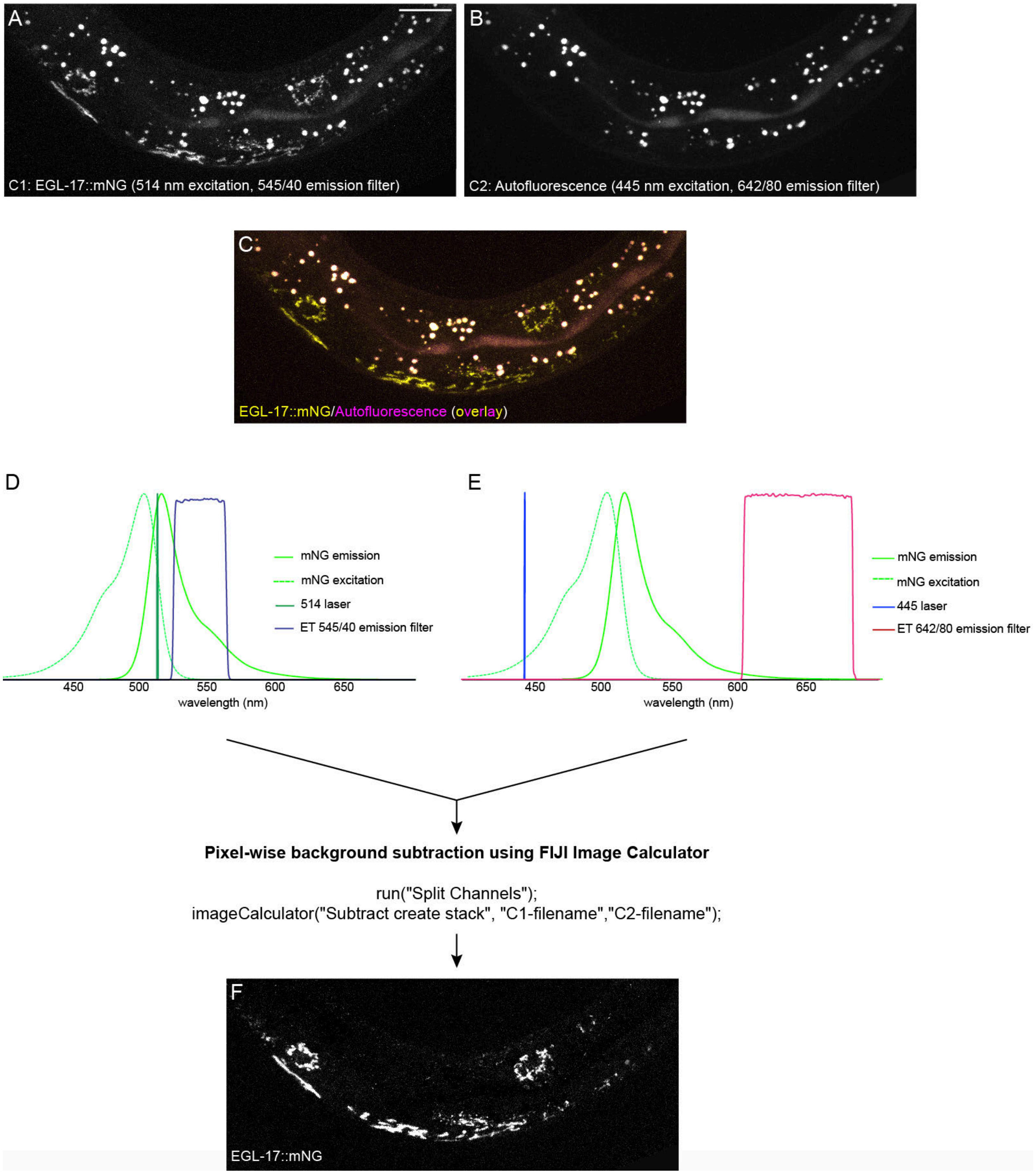

Fig. S3. Workflow for autofluorescence subtraction.
To subtract broad-spectrum autofluorescence, we acquired paired fluorescent protein **(A)** and background **(B)** channels. **(C)** Merged image showing overlap between channels. Note that gut granules are visible in both channels (indicated by white in overlay) whereas FGF/EGL-17::mNG (yellow) fluorescence is not visible in the background channel. **(D, E)** Schematic diagrams of mNG excitation/emission spectra and the optical configurations used for imaging mNG **(D)** or autofluorescence **(E)**. **(F)** Subtracting the autofluorescence channel from the fluorescent protein channel using the Fiji Image Calculator removes gut granules and enhances the ability to visualize FGF/EGL-17::mNG, which is expressed at low levels. Scale bar = 10 mm.

Development • Supplementary information

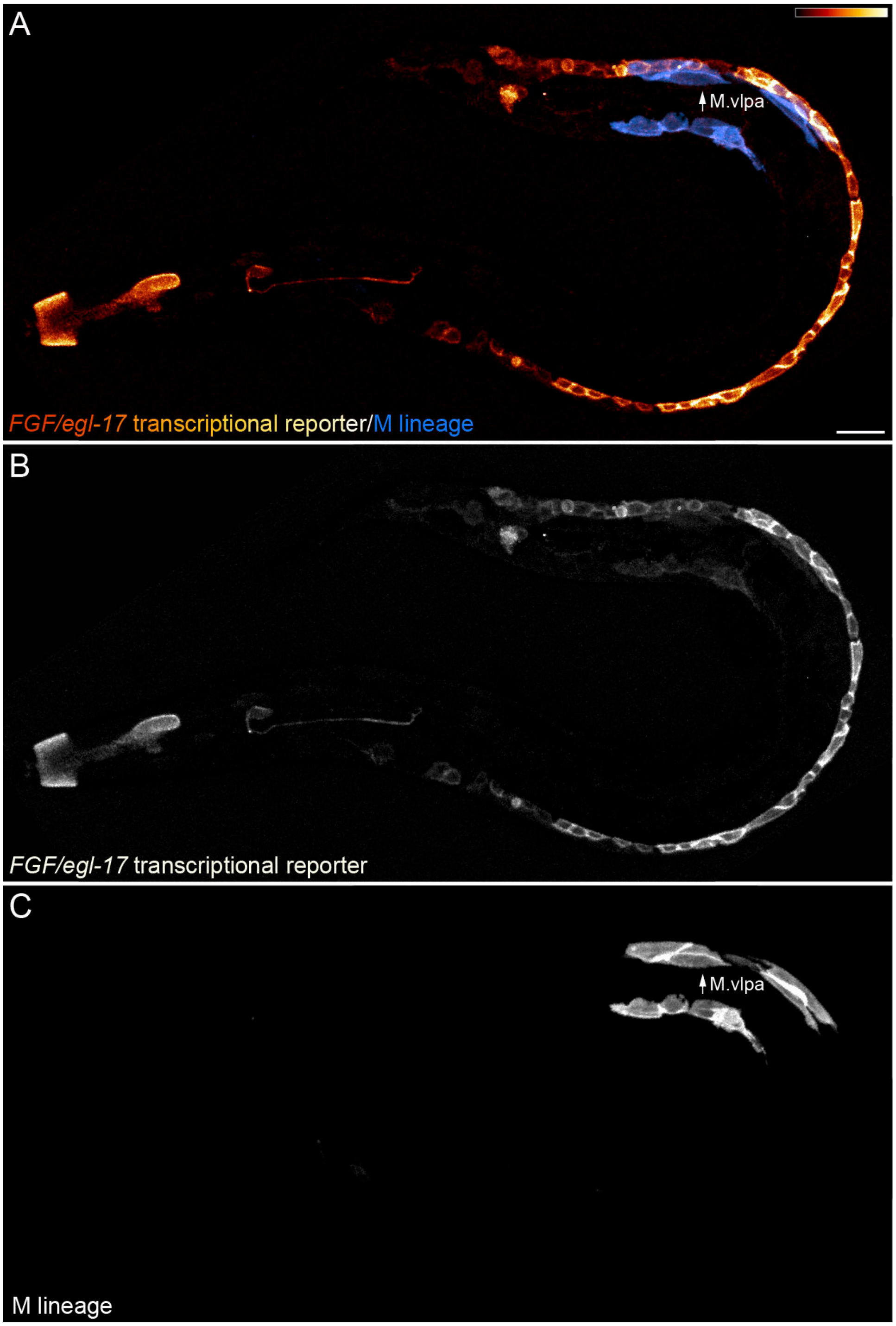

Fig. S4. Endogenous *FGF/egl-17* transcriptional reporter expression prior to SM birth. (A-C) Images show an entire larval animal with highlighting *FGF/egl-17::SL2::mNG::PH* transcriptional reporter expression along the ventral midline. *FGF/egl-17* is also weakly expressed in M lineage cells at this time point . The SMs are born from an asymmetric division of M.vlpa on the left side (arrow in A, C) and M.vrpa on the right side (not shown). Images are maximum intensity projections of spinning disk confocal slices after autofluorescence background subtraction (see Fig. S3). Animal is oriented with head to the left. The *FGF/egl-17* transcriptional reporter is expressed in along the ventral midline. Scale bar = 10 mm.

Development • Supplementary information

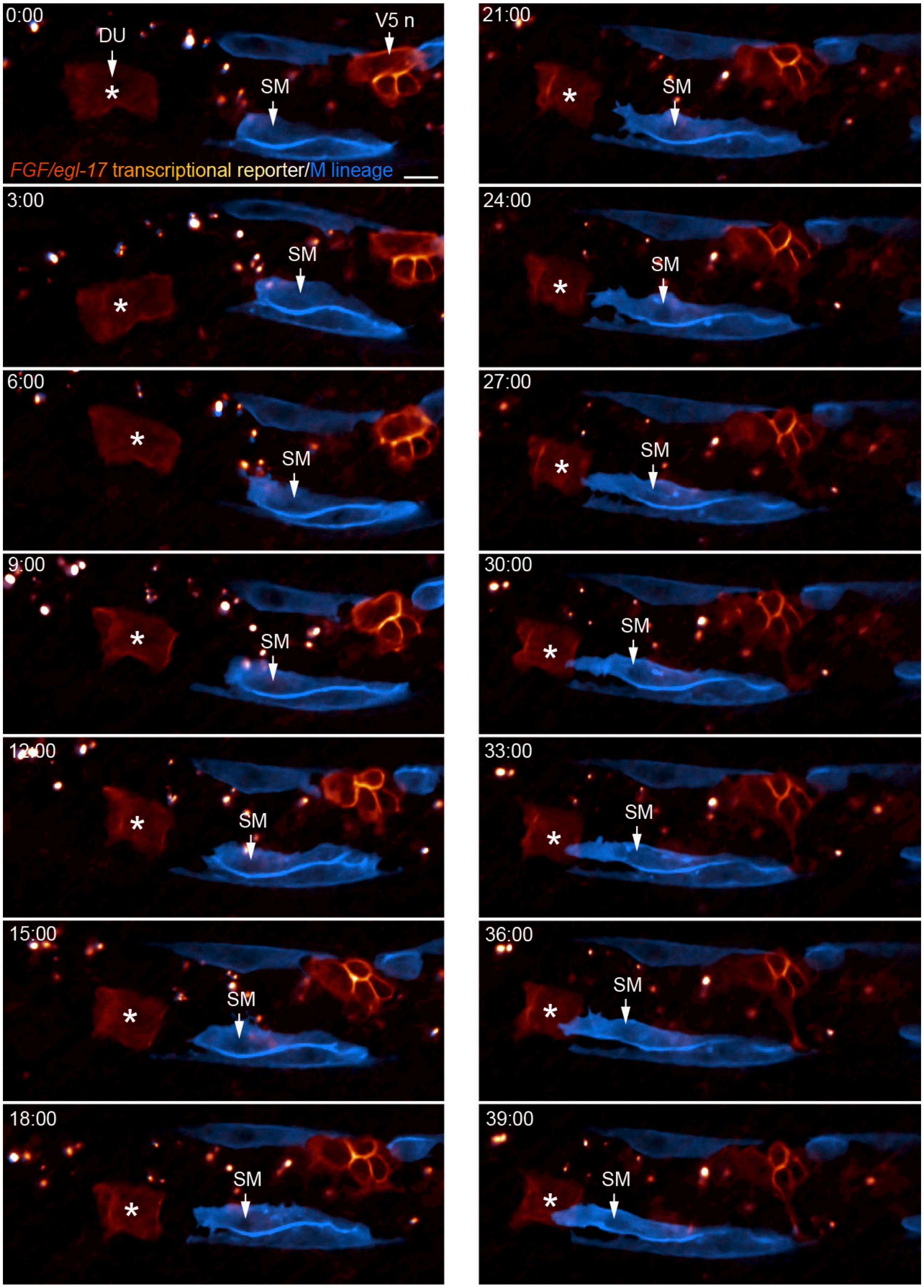

Fig. S5. Time-lapse imaging of migrating SM and *FGF/egl-17* expressing cells during FGF-dependent migration. Images show single frames from Movie 2. Time is indicated in minutes::seconds. Asterisk marks the migration endpoint over the *FGF/egl-17*-expressing DU cell. Abbreviations: DU, dorsal uterine cells; SM, sex myoblast; V5 n, V5-derived neuroblasts and neurons. Animal is oriented with anterior to left and dorsal to top. Scale bar = 10 mm.

Development • Supplementary information

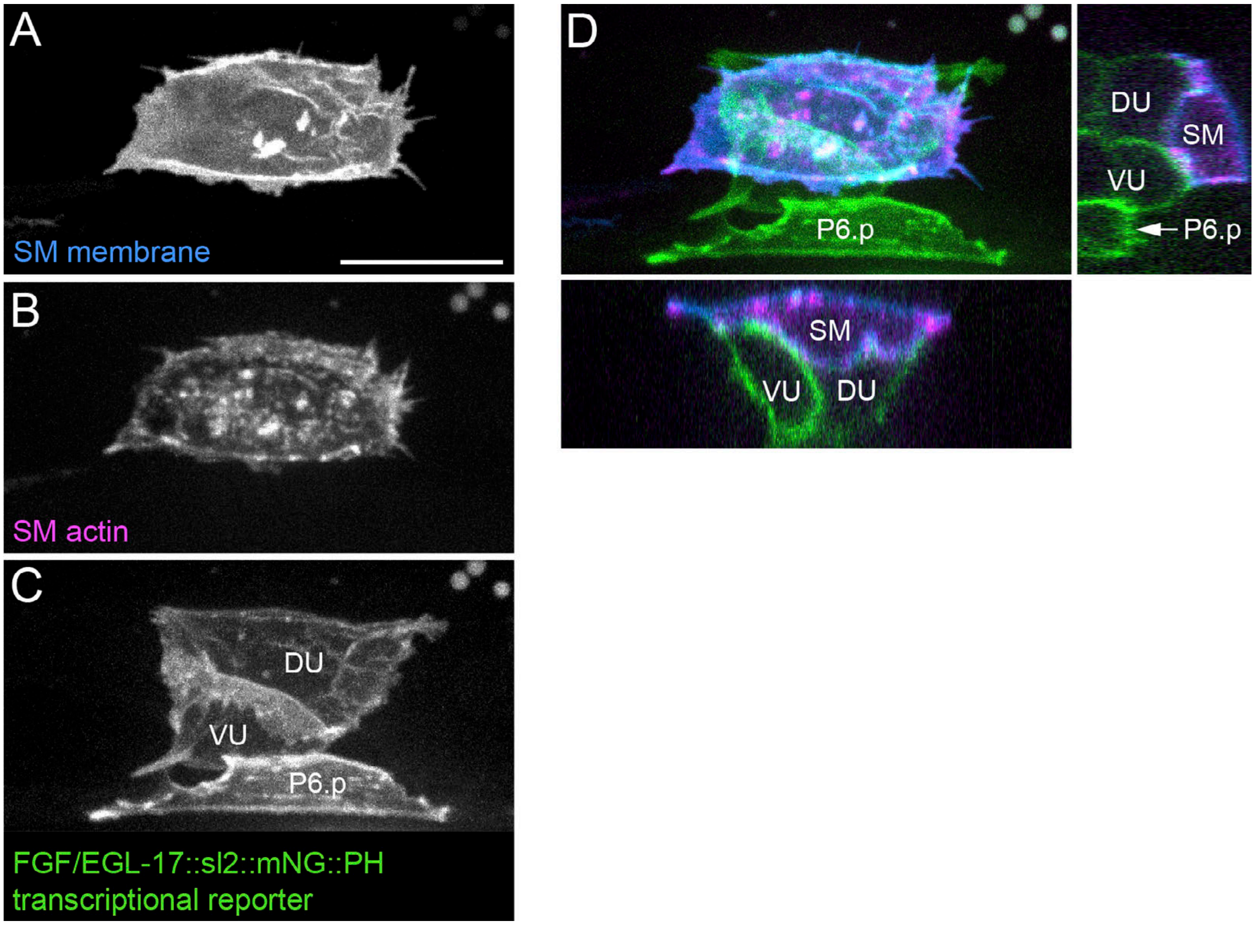

Fig. S6. Final SM positioning over the uterine and P6.p cells. Images show maximum intensity projections of the SM membrane **(A)**, actin **(B)**, and membranes of *FGF/egl-17*-expressing cells **(C)**. **(D)** Merged image with orthogonal projections showing the SM final position directly over the uterine cells. Abbreviations: DU, dorsal uterine cell; SM, sex myoblast; VU, ventral uterine cell. Animal is oriented with anterior to left and dorsal to top. Scale bar = 10 mm.

Development • Supplementary information

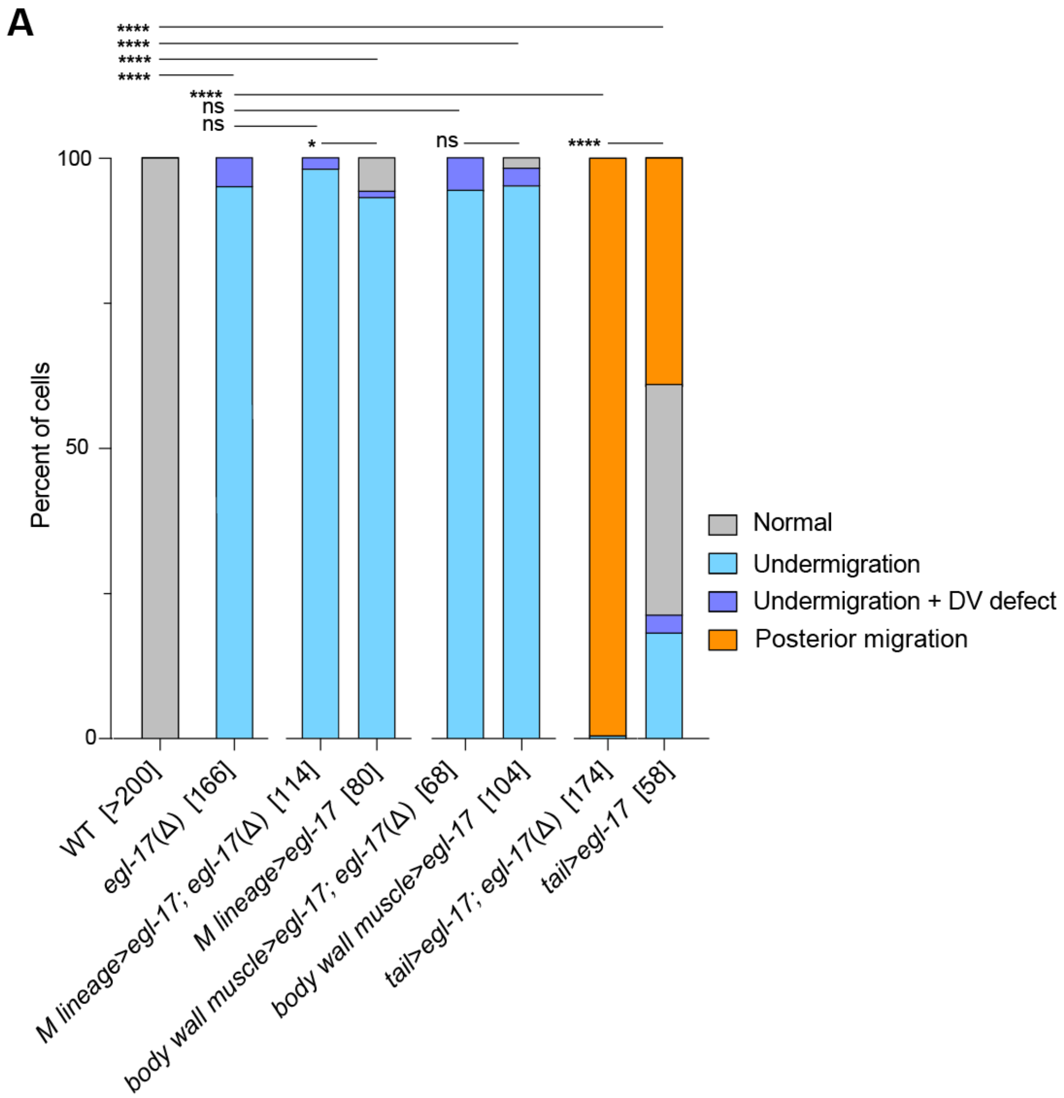

B

Strain	*FGF/egl-17* expression [n]	normal migration	undermigration	undermigration + dorsoventral defect	posterior migration
APL5	WT [>200]	100	0	0	0
APL299	*egl-17*(Δ) [166]	0	96	4	0
APL201	*M lineage>egl-17; egl-17*(Δ) [114]	0	98	2	0
APL200	*M lineage>egl-17* [80]	6	93	1	0
APL126	*body wall muscle>egl-17; egl-17*(Δ) [68]	0	94	6	0
APL342	*body wall muscle>egl-17* [104]	2	95	3	0
APL242	*tail>egl-17; egl-17*(Δ) [174]	0	1	0	99
APL316	*tail>egl-17* [58]	40	16	3	41

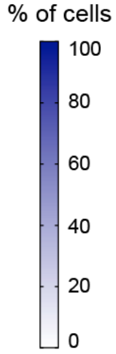

Fig. S7. FGF misexpression interferes with SM migration in a wild-type background and fails to rescue SM migration in *FGF/egl-17(Δ)* animals. (A) Chart showing the percent of SMs with abnormal migration phenotypes in misexpression experiments. Significances of the differences in phenotypic frequency distributions between strains were assessed using pairwise Fisher's Exact Tests. **** P<0.0001, * P=0.011, ns=not significant (P>0.05). **(B)** Data used to construct chart in A. Opacity indicates the percent of cells showing each migration phenotype. The number of cells examined (2 per animal) is indicated in brackets.

Development • Supplementary information

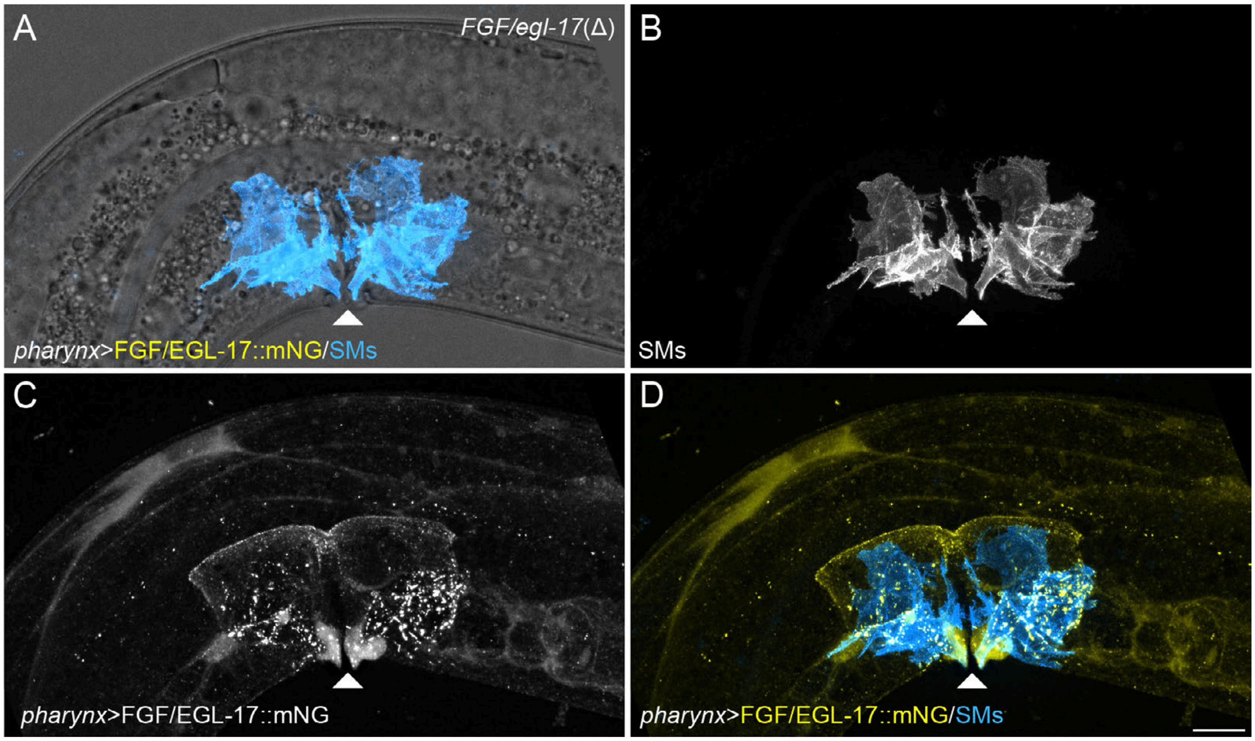

Fig. S8. Precise SM positioning and normal muscle subtype differentiation in a *FGF/egl-17(Δ)* + *Pmyo-2*>FGF/EGL-17::mNG animal at the L4 stage. **(A)** SM descendants are precisely positioned flanking the vulva despite the absence of local *FGF/egl-17* expression. **(B)** Egg-laying muscles exhibit normal morphology. **(C, D)** FGF/EGL-17::mNG disperses from the head to the SMs and somatic gonad cells. The smooth, hazy signal prominent on the top left represents FGF/EGL-17::mNG protein in the extracellular space. Animal is oriented with anterior to left and dorsal to top. White triangles denote the vulva. Scale bar = 10 mm.

Development • Supplementary information

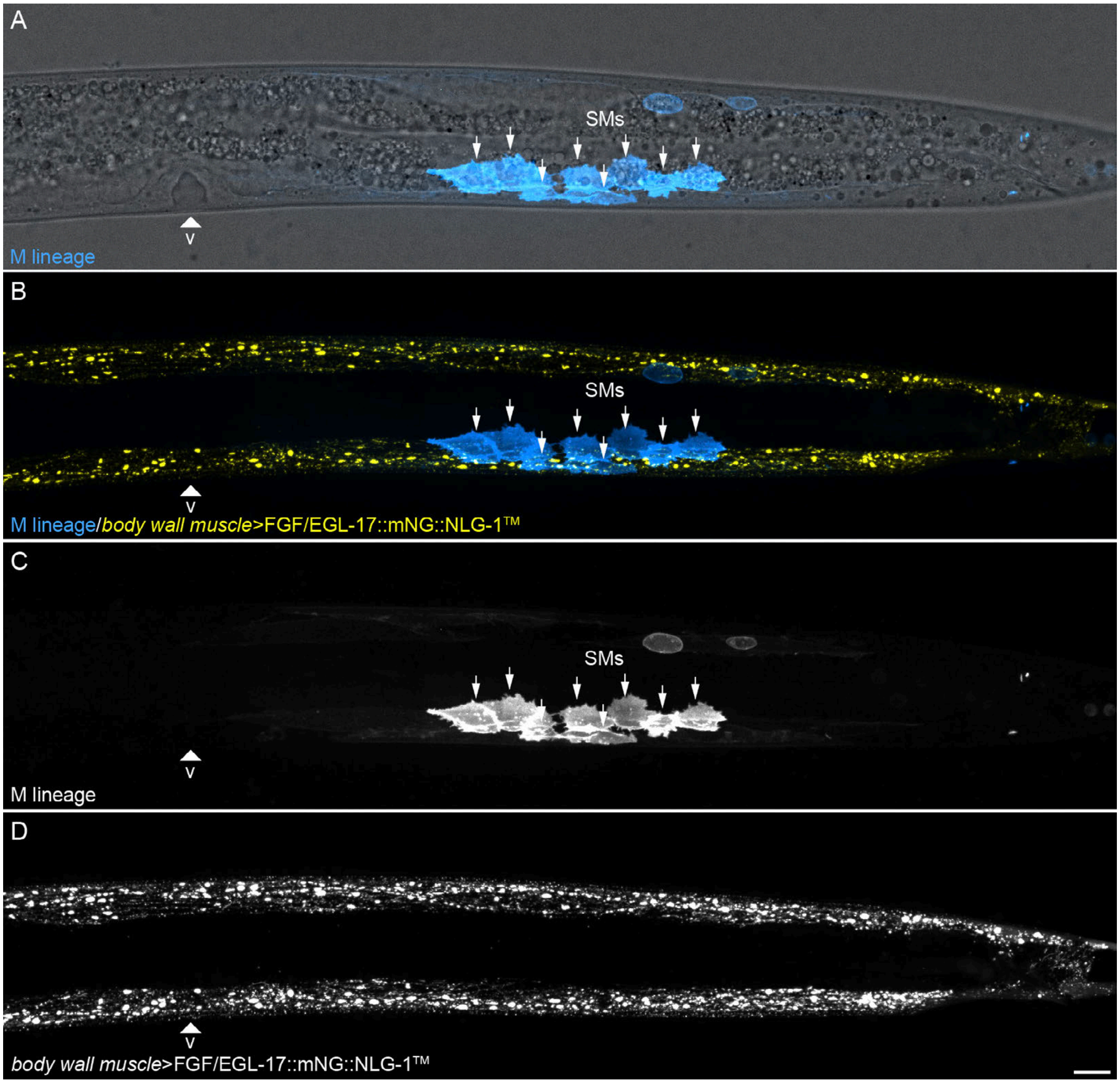

Fig. S9. Membrane-tethered FGF/EGL-17::mNG::NLG-1^{TM} is capable of contact-dependent signaling. Expressing membrane-tethered FGF/EGL-17 in body wall muscles arrests SM migration and phenocopies diffusible FGF expression in the same cell type. Images show a larval animal at the 8 SM stage after divisions are complete. **(A)** Overlay of M lineage cells and transmitted light image to show morphology. **(B)** Merged image of M lineage cell membranes **(C)** and FGF/EGL-17::mNG::NLG-1^{TM} **(D)**. Animal oriented with anterior to left and dorsal to top. White triangles denote the vulva (abbreviated by v). Scale bar = 10 mm.

Development • Supplementary information

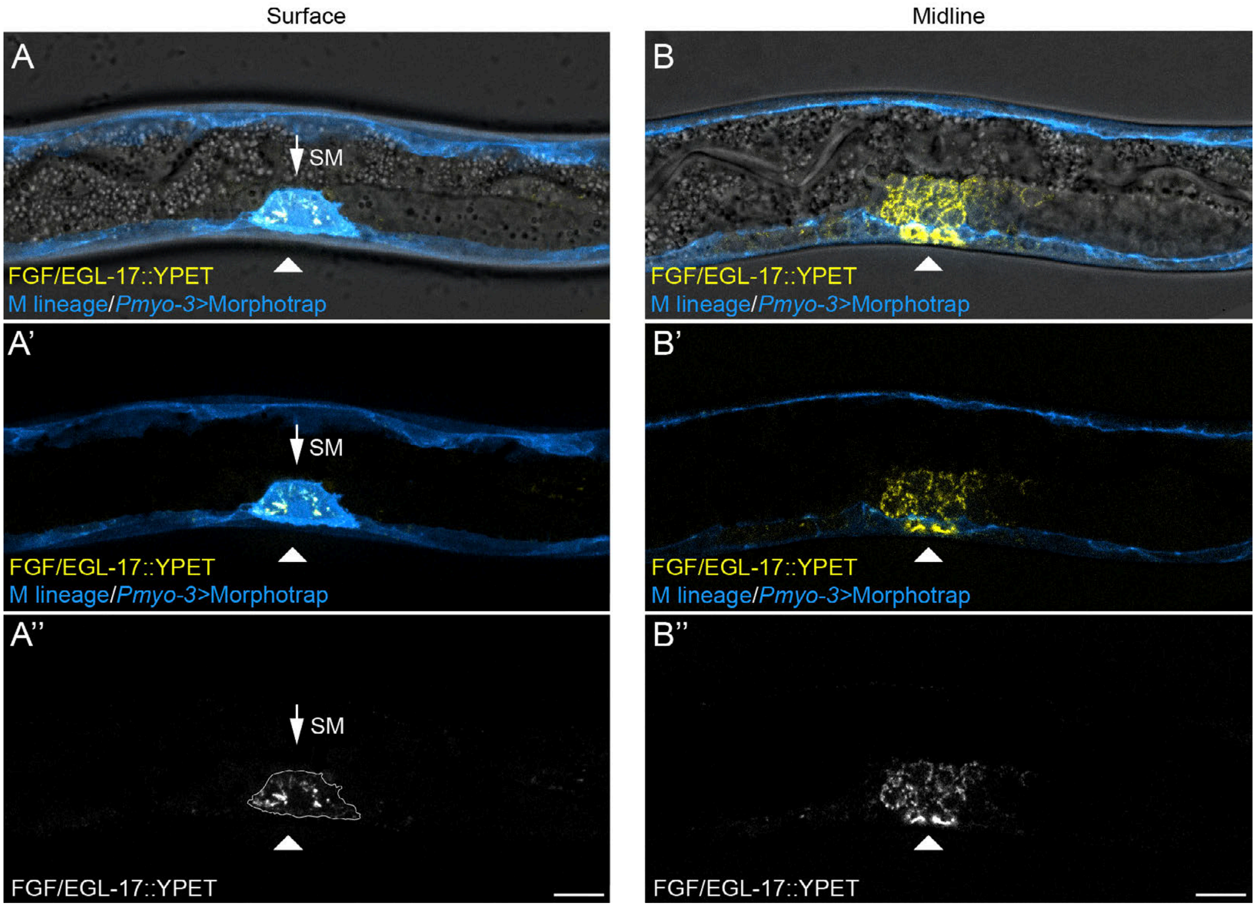

Fig. S10. Morphotrap does not sequester FGF/EGL-17::YPET. Surface **(A)** and midline **(B)** planes showing endogenously tagged FGF/EGL-17::YPET, the M lineage, and *Pmyo-3*>Morphotrap. Note that FGF/EGL-17::YPET localizes to the SM, but not to Morphotrap-expressing body wall muscles. Animal oriented with anterior to left and dorsal to top. White triangles denote the normal SM migration endpoint over P6.p. Scale bar = 10 mm.

Development • Supplementary information

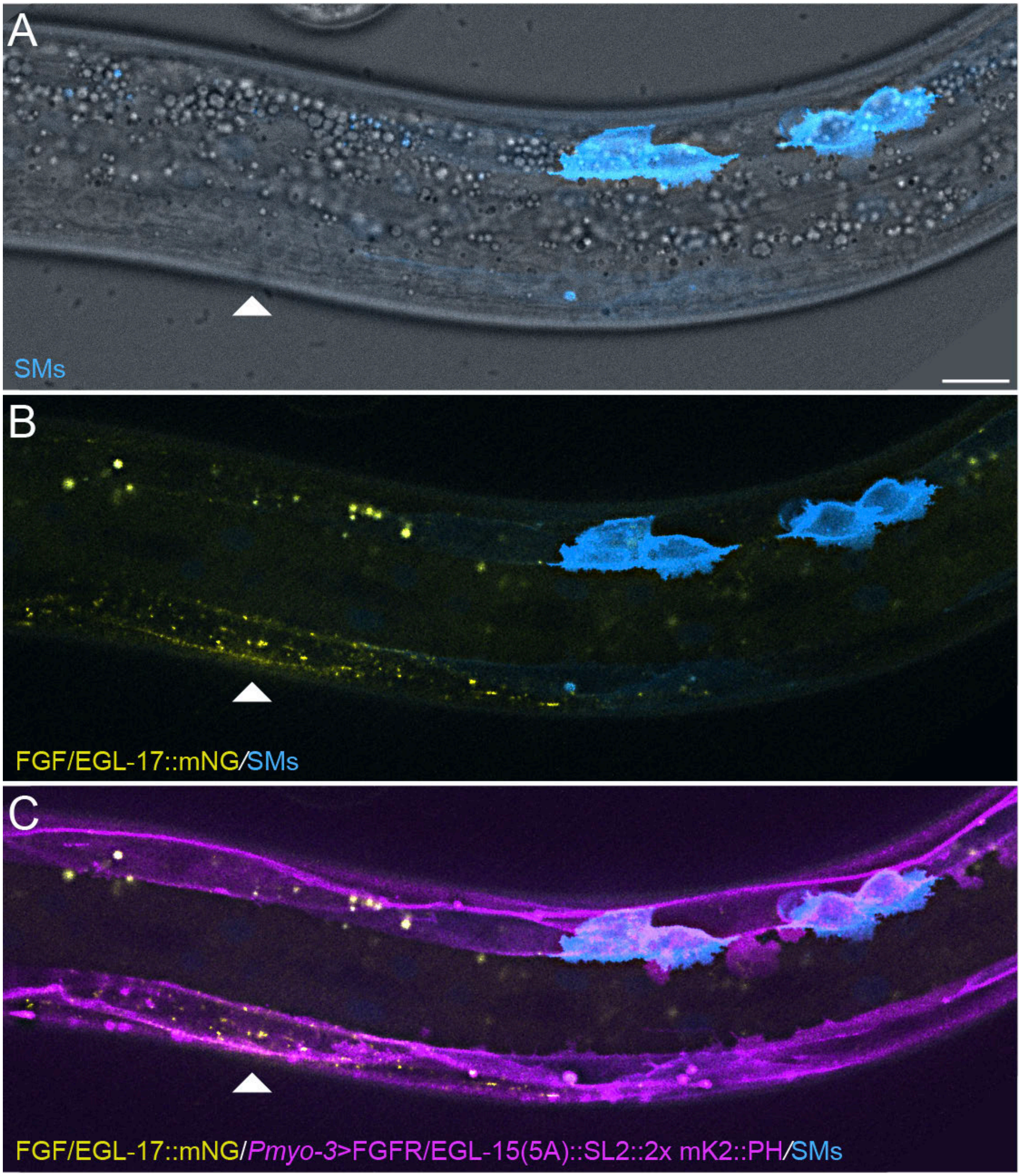

Fig. S11. Dorsoventral and anteroposterior migration defects in a *Pmyo-3>FGFR/egl-15(5a)::SL2::2x mKate2::PH* animal. **(A)** Overlay of fluorescence and transmitted light images showing dorsally and posteriorly displaced SMs in an animal with FGFR/egl-15(5a) expression in body wall muscles. **(B, C)** Endogenously tagged FGF/EGL-17::mNG localizes to body wall muscles expressing FGFR/egl-15(5a), which sequester extracellular FGF and limits its availability to SMs. Note the lack of FGF/EGL-17::mNG fluorescence in the SMs. Animal oriented with anterior to left and dorsal to top. White triangles denote the normal SM migration endpoint over P6.p. Scale bar = 10 mm.

Development • Supplementary information

Table S1. Source data for SM migration phenotypes

Strain	Experimental manipulation [n]	overmigration	overmigration + dorsoventral defect	normal migration	undermigration	undermigration + dorsoventral defect	dorsoventral defect	posterior migration
APL5	WT [>200]	0	0	100	0	0	0	0
APL299	*egl-17*(Δ) [166]	0	0	0	96	4	0	0
APL201	*M lineage>egl-17; egl-17*(Δ) [114]	0	0	0	98	2	0	0
APL200	*M lineage>egl-17* [80]	0	0	6	93	1	0	0
APL126	*body wall muscle>egl-17; egl-17*(Δ) [68]	0	0	0	94	6	0	0
APL342	*body wall muscle>egl-17* [104]	0	0	2	95	3	0	0
APL242	*tail>egl-17; egl-17*(Δ) [174]	0	0		1	0	0	99
APL316	*tail>egl-17* [58]	0	0	40	16	3	0	41
APL45	*pharynx>egl-17; egl-17*(Δ) [92]	41	1	39	19	0	0	0
APL130	*membrane-anchored egl-17* [162]	1	0	0	95	4	0	0
APL243	*body wall muscle>membrane-anchored egl-17* [98]	0	0	0	100	0	0	0
APL670	*body wall muscle>Morphotrap; egl-17::YPET* [76]	0	0	100	0	0	0	0
APL622	*body wall muscle>FGFR/egl-15(5A)* [194]	0	0	48	44	6	2	0

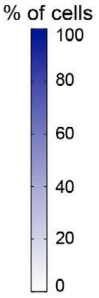

Development • Supplementary information

Table S2. Resources and reagents

Resource or Reagent	Source
***C. elegans* strains**	
C. elegans: APL5: ljfSi2[Phlh-8>mKate2::*D. melanogaster* moesin actin-binding domain::F2A::2x mTurquoise2::PH::3xHA::tbb-2 3'UTR loxN ttTi4348] I	This paper
C. elegans: APL23: ljfSi10[Phlh-8>2x mTurquoise2::PH::tbb-2 3'UTR loxN ttTi5605] II; egl-17(ljf7[egl-17::mNG^3xFlag]) X	This paper
C. elegans: APL45: ljfSi18[Pmyo-2>egl-17::mNG::tbb-2 3'UTR loxN ttTi4348] I; ljfSi10[Phlh-8>2x mTurquoise2::PH::tbb-2 3'UTR loxN ttTi5605] II; egl-17(ljf14[deletion + mNG^3xFlag]) X	This paper
C. elegans: APL126: ljfSi33[Pmyo3>egl-17::mNG::SL2::2x mKate2::PH::3xHA::tbb-2 3'UTR loxN ttTi4348] I; ljfSi10[Phlh-8>2x mTurquoise2::PH::tbb-2 3'UTR loxN ttTi5605] II; egl-17(ljf14[deletion + mNG^3xFlag]) X	This paper
C. elegans: APL130: ljfSi10[Phlh-8>2x mTurquoise2::PH::tbb-2 3'UTR loxN ttTi5605] II; egl-17(ljf25[egl-17::mNG::nlg-1 C-terminus]) X	This paper
C. elegans: APL199: ljfSi10[Phlh-8>2x mTurquoise::PH::3xHA::tbb-2 3'UTR loxN ttTi5605] II; egl-17(ljf24[egl-17::SL2::mNG::PH]) X	This paper
C. elegans: APL200: ljfSi34[Phlh-8>egl-17::tbb-2 3'UTR loxN ttTi4348]) I; ljfSi10[Phlh-8>2x mTurquoise2::PH::tbb-2 3'UTR loxN ttTi5605] II	This paper
C. elegans: APL201: ljfSi35[Phlh-8>egl-17::tbb-2 3'UTR loxN ttTi4348]) I; ljfSi10[Phlh-8>2x mTurquoise2::PH::tbb-2 3'UTR loxN ttTi5605] II; egl-17(ljf14[deletion + mNG^3xFlag]) X	This paper
C. elegans: APL242: ljfSi32[Pegl-20(-1261 – 610)::pes-10>egl-17::mNG::SL2::2x mKate2::PH::3xHA::tbb-2 3'UTR loxN ttTi4348]) I; ljfSi10[Phlh-8>2x mTurquoise2::PH::tbb-2 3'UTR loxN ttTi5605] II; egl-17(ljf14[deletion + mNG^3xFlag]) X	This paper
C. elegans: APL243: ljfSi36 [Pmyo3>egl-17::mNG::nlg-1 C-terminus::tbb-2 3'UTR loxN ttTi4348] I; ljfSi10 [Phlh-8>2x mTurquoise2::PH::tbb-2 3'UTR loxN ttTi5605] II	This paper
C. elegans: APL299: ljfSi2 [Phlh-8>mKate2::*D. melanogaster* moesin actin-binding domain::F2A::2x mTurquoise2::PH::3xHA::tbb-2 3'UTR loxN ttTi4348] I; egl-17(ljf14[deletion + mNG^3xFlag]) X	This paper
C. elegans: APL311: ljfSi41 [Phlh-8>2x mTurquoise2::PH::3x HA::tbb-2 3'UTR loxN ttTi4348] I; ljfSi42 [egl-17>2x mKate2::PH::3xHA::tbb-2 3'UTR loxN ttTi5605] II; arTi133 [Phlh-8>ERK-nKTR::mClover::T2A::mCherry::H2B::unc-54 3'UTR]	This paper; De la Cova, et al., 2017
C. elegans: APL319: ljfSi37 [Pegl-20(-1261 – 610)::pes-10>egl-17::mNG::SL2::2x mKate2::PH::3xHA::tbb-2 3'UTR loxN ttTi4348] I; ljfSi10 [Phlh-8>2x mTurquoise2::PH::tbb-2 3'UTR loxN ttTi5605] II	This paper
C. elegans: APL342: ljfSi38 [Pmyo3>egl-17::mNG::SL2::2x mKate2::PH::3xHA::tbb-2 3'UTR loxN ttTi4348] I; ljfSi10 [Phlh-8>2x mTurquoise2::PH::tbb-2 3'UTR loxN ttTi5605] II	This paper
C. elegans: APL622: ljfSi2 [Phlh-8>mKate2::D. melanogaster moesin actin-binding domain::F2A::2x mTurquoise2::PH::3xHA::tbb-2 3'UTR loxN ttTi4348] I; ljfSi39 [Pmyo-3>egl-15(5a)::SL2::2x mKate2::PH::3xHA::tbb-2 3' UTR lox511i] IV; egl-17(ljf7[egl-17::mNG^3xFlag]) X	This paper
C. elegans: APL670: ljfSi40 [Pmyo-3>pat-3 signal peptide::2x vhhGFP4::CD8 transmembrane domain::2x mTurquoise2::PH::tbb-2 3'UTR loxN ttTi4348] I; ljfSi10 [Phlh-8>2x mTurquoise2::PH::tbb-2 3'UTR loxN ttTi5605] II; egl-17(ljf27[egl-17::YPET^3xFlag]) X	This paper, Pani & Goldstein, 2018
C. elegans: N2: wild-type	CGC
**Plasmids**	
Plasmid: pAP082: Peft-3>Cas9 + PU6>Chr I (ttTi4348) sgRNA	Pani & Goldstein, 2018
Plasmid: pAP087: 2x mKate2::PH::3xHA::tbb-2 3'UTR loxN SEC loxN backbone for cloning transgenes, Chr II	Pani & Goldstein, 2018
Plasmid: pAP088: 2x mTurquoise2::PH::3xHA::tbb-2 3'UTR loxN SEC loxN backbone for cloning transgenes, Chr I	Pani & Goldstein, 2018
Plasmid: pAP092 Pegl-17>2x mKate2::PH::3xHA::tbb-2 3'UTR loxN SEC loxN, Chr II	This paper

Development • Supplementary information

Plasmid: pAP137 Phlh-8>2x mTurquoise2::PH::3xHA::tbb-2 3'UTR loxN SEC loxN, Chr II	This paper
Plasmid: pAP138 Phlh-8>2x mTurquoise2::PH::3xHA::tbb-2 3'UTR loxN SEC loxN, Chr I	This paper
Plasmid: pDD122: Peft-3>Cas9 + PU6>Chr II (ttTi5605) sgRNA, Addgene #47550	Dickinson, et al., 2013
Plasmid: pDD162: Peft-3>Cas9 + PU6>empty sgRNA for cloning, Addgene #47549	Dickinson, et al., 2013
Plasmid: pDD268: mNG^SEC^3xFlag backbone for cloning repair templates, Addgene #132523	Dickinson, et al., 2015
Plasmid: pDD283: YPET^SEC^3xFlag backbone for cloning repair templates, Addgene #66824	Dickinson, et al., 2015
Plasmid: pDD315: mTurquoise2^SEC^2xHA backbone for cloning repair templates, Addgene #73343	Dickinson, et al., 2015
Plasmid: pRCA5: Phlh-8>mKate2::Dme moesin ABD::F2A::2x mTurquoise2::PH::3xHA::tbb-2 3'UTR loxN SEC loxN, Chr I	This paper
Plasmid: pTG42: egl-17::mNG^SEC^3xFlag::nlg-1 C-terminus homologous repair template	This paper
Plasmid: pTG003: egl-17::mNG^SEC^3xFlag homologous repair template	This paper
Plasmid: pTG004: Peft-3>Cas9 + PU6>egl-17 C-terminus sgRNA1	This paper
Plasmid: pTG006: egl-17::YPET^SEC^3xFlag homologous repair template	This paper
Plasmid: pTG009: Peft-3>Cas9 + PU6>egl-17 C-terminus sgRNA2	This paper
Plasmid: pTG012: Peft-3>Cas9 + PU6>egl-17 N-terminus sgRNA for deletion	This paper
Plasmid: pTG034: egl-17 deletion + mNG^SEC^3xFlag homologous repair template	This paper
Plasmid: pTG052: egl-17::SL2::mNG::PH^SEC^ homologous repair template	This paper
Plasmid: pTG077: Pmyo-2>egl-17::mNG::tbb-2 3'UTR loxN SEC loxN , Chr I	This paper
Plasmid: pTG110: Peft-3>Cas9 + PU6>Chr IV sgRNA	This paper
Plasmid: pTG125: Pmyo3>egl-17::mNG::SL2::2x mKate2::PH::3xHA::tbb-2 3'UTR loxN SEC loxN, Chr I	This paper
Plasmid: pTG238: Phlh-8>egl-17::mNG::tbb-2 3'UTR lox511i SEC lox511i , Chr IV	This paper
Plasmid: pTG282: Pegl-20(-1261 – 610)::pes-10>egl-17::mNG::SL2::2x mKate2::PH::tbb-2 3'UTR lox 511i SEC lox 511i, Chr IV	This paper
Plasmid: pTG284: Pmyo-3>egl-15(5A)::SL2::2x mKate2::PH::tbb-2 3' UTR lox511i SEC lox 511i, Chr IV	This paper
Plasmid: pTG327: Pmyo3>egl-17::mNG::nlg-1 C-terminus::tbb-2 3'UTR lox511i SEC lox511i , Chr IV	This paper
**Key Reagents**	
Gibco Hygromycin B (50 mg/mL) (Fisher Scientific)	10-687-010
NEBuilder HiFi DNA Assembly Cloning Kit (New England Biolabs)	E5520S
Invitrogen PureLink HQ Mini Plasmid DNA Purification Kit	K210001
Q5 High-Fidelity 2X Master Mix (New England Biolabs)	M0492L
Q5 Site-Directed Mutagenesis Kit (New England Biolabs)	E0554S

Development • Supplementary information

Supplementary Materials and Methods

Molecular cloning information

This file includes sequence information for transgene promoters used in this study, the *nlg-1* C-terminal region used for membrane tethering, and a Chr IV transgene insertion site that has not been described elsewhere.

egl-20$^{(-1261 – 610)}$ enhancer:

*Underlined sequence is the *pes-10* minimal promoter.

gttggatgttttcttataaaattatcttggaagtttttttaaaaattaaatactctgttacatttttttgcaatgttt
ggaattgaaatatagaacaatataaaacttctagagagaaaaattacttccagaatattgataaatgataaagaata
ttgataacctgaggatttgccacttttgacggcagcattaaaaattgcaattggatactcaagaagataaagtgat
caattatggagaaaataaaatactgtgtaaataaatgattttttaaacattttttccgaaatataggtgttccat
ttgtcacatgataattatgaatattcttgaatttctatgttttgaaaaattgaagctaaaaaataagtgtcaaatat
tgttaaagtcccaaaaatcttggaatttttcgtttcgtattgaccattcacttcttcccatccttttcaccatttc
aacgtcttcctcttctatttgcataccgacaacgtcttcttctcttccattttctgaatagatccacgggccataaa
gcatgaattggaaaggatggagaagatgacaagataccacatccagcaccatcaattcgaccttcgaattttctaaa
cacattgacattcctcttattcatcattcttcatccctgcaggatcgatttttttgcaaattacgagcgttgtagggg
gcggagcgataggtcctataggttttggtatatcatcattcattcattcattggtacattcatttacccaccttcct
ctttctgagcttctctggagttctgtgcttcctttttcccttatctttatactgtaatttttaactttcaggcattg
attggatcattggccaaaggacccaaaggtatgtttcgaatgatactaacataacatagaacattttcaggaggacc
cttgcttggaggagctcagaaaa

***hlh-8* promoter:**

aaagctttcaaaatcccaaacatagcatcttacttactcgcctttgctcccaaagatttaccattttcttgtttttg
tttgttaatttcctctcccacccattttcaaccatgtcatttcacacattaaactcagtatgcatcgatttaattgt
tccaggctaattgggtttctgtcattgttaatagacacaatttttactgagaaatccttttcatatgtgtgaaattt
gttctttaaacgtgcatttcctatttatcgctctctagatttgatgccagttcgtgtagtttttttcccaaccgttga
ctcatcgataccgcaatccatttcacccagtatttgtaccattgccgtgaattcacagtattacatggtcgtcccct
tttccacaaaatctctttcagactccctccaccctgatcccgttttaattgtttcttgctgatttccctttcaaccg
atcttcgtttttttttcaatagggtatatttgaaatgtcggaatgtcgattcagcagtaaatttgatatgacactct
gactttttaaatgtaaatctgattgtttatcagaattcaaccgggcccactgattattcattaattatgcgacaag
aagaataacgttatagtttttctttcttaagcatttttcatcatcaacatttttttgtttagatttaattttgttgct
aataaaaaactacacaaaacgccaagcacatttatttgactttttttttttgagtaatgcagtaagtaggcaatttgt
tgtttttataattgattagttttaaggcctacttgcaaaactgagaaaattcttatcaagaattcaaaaccatttac
gattatcggattttaatatggacaatgttactcattttggataaatcaaaagagtttttggattttaaaattaata
actgtttattactgattaacaaaagattacggtaaattggagttacaaaatggacaagttttatttatgaatcaaga
catattttgataaaaaatatgggtacccccgccaatttaaaaataaattttttaacttaaaaaaaaacgtgtgaaat
gctttcatatttcaggcttacttagaaaaacaccattcctaagtctaacgaggaaaatgggaaacatgggaaatat
taccgaaacctgggaaatatttttttattgattccaaattttcccttgattccaaatatcgatgtgaaaaaaaattaa
aacaaaaattactgattttatttaagcttgaaatcacaaattccatttttatgtcatacttcagatttaaacaaatt
tattttatgtgtgttttttttaatggtatgtcctaacgattttcctaattgacaactattatagattgaaaacacaga
atgccaagtaacgtaagaaatttttttttcgaataccagacataatttcaataaaagaaaaatctttaaaaaaagtt
taactttatactacaataattttggtttttatgaaggaatctgtattgcggcacatcatgtatttgctcaaggtttc
gttggttgacatatttttgtgatccgtggaatgagcttgcatagagtttagtcataattgggtttattttacataca
gtttgaagtatgttttgtctgttattcgaaatttttttagttagtttggtaagttgaaaagaatttacaaaatttaac
taaacgaggacgctcatctcgattcttataattccataataattctacggtaaaagtcaagttatgcctcaaaacat
gtaatacataattaccaaaactacttaattaccgcatttccttagtattttaatagtggtgtagtcgaattttttt
attgctttattagactcaaaattgtctgcaaacacccaatttcataatgaaacttattgaaaacaatacactttgaa
acaatttagtatttcaggaataaggtcactggaacttcgaaaatgataatttgaataaccgttaataaaatattcaa
accaatttgcataatcttgattttgtatcatgatggtatagaatgggacttttgaaagatgtgaagtttcaattcag
taagtacaactttaaaattgggctgcaagatttcttcttaaattttcaacagtttcaaaacactgaaatcatgctga
actatggttagagactcgacaactttcctaaattttggagcagtttcaatggttttcgaatgtatactacatttgta
ccattttatgtgttagtgtaggttgctttgcttctaccttcactctcaaattctttttttccgcggtaatttttcaac
tatcgaaacaaagaagaagtcatgaataactgtccgcatagagttctcacccgtccccttttcttttcaccgagcagt
cacgagagaaagaagaagaagcggaccgctgcagagattcttcgtagcggagccccgcccatcctctgttacgcata
tgtttggatctcatttcttccactcattaacgttccaggtatgtttttttttgttgatttttttaatgtgttacttttc
atatgtttattacttgaactgaccgatttcaaatatgattttcacag

Development • Supplementary information

myo-3 promoter:

cggctataataagttcttgaataaaataattttcccgacaaaacatgagtatttctttcgaaaataaaagtgcaggc
taattagagattattctgtaattaactgcataatttgtcacgtgccatagttttacattccactacgtcatagttct
taaaatactaatctcctgaaaatagaagtaggtgaagaaagtttaattatcagttctaaaatgacaattgatctttg
gaatatgttctgaaactaccgatcattgaacagatgctatttgaatgatatagaattgtatatttgcaatttctgaa
acgcgttcttaaaggcacacagattaattcaaaagggtctggccgcaaaaaggtttatggtggccgattttgagttt
tgtgtgtgattgctttttcacaatcagtgttttcaggattatgtgatgaactagatcttcaagtttcgttacatttc
atatgttttcggaactcacgaagtacatattgggtattgtgctcaaaaaattcagcaatcagcttcgctccgctgac
tttagaacccaaaaaaatagtatggccaaactgactgtgttacgatcatttcaattttcaatacatatttaagatt
tctaagagtaagaaggtcaaaaactgttctggaatacatatatatttttcaggttacaattagtcaaaaagtgcact
gaaatatacgttttaatttcacgaataacccaattagttcaatgtatttttggtcaaccaacgttaaagtttggctt
ccaaccaattatcatttctgatcaaccacaatgtttttctttatctgcaagttaatttttattttatccagatg
tttggcatatttttcaattcttcactagcgcccacttcttgcacttccggcgccctgaatctaatgcatctgttgca
agaattgaaagaccaatcaacacattgttttcttcacgagatactgaagaaaatgaataaaaacagagaaaaagagc
catgtgattagtgacaactgttgctaacagataccatagcttggacttggtacgtgatggcaacgtatgggtcaaca
aaaatgattgcagagggggtgcaaaacagtcaagtcgagaaaatatgaaaaacagaaaacaaagaacagaaaaatgg
gtttgagagtcagtataatttataaaagaaaaattgtacatagaaattaaccattttgtagaagaagttatttttc
aagcatcgttaaaaattattcaaagcaccttatttcatatttaattttaaacatggttaaatgaacaacacggtgcg
caatcaggaaaacttgaaatctgaaactgttgttgtgatcttcttcgcaactgttcagatagcactagtgtaatgtt
aagagtgcgcgaatataatggaatataatggatcacacctcctgccatcaggtaaacgtctctgttatcacatattt
ccaactattaaattttacctttacagttttacatttttttgaaaaaagtaacttttgtcttcaaaatccctgac
gaaaatatcaaatatttaatcgagactgcagaggaaccgattgatgatttggaaaatccagctttacctgtgtaag
aactgaaaagtttcataaccctagggtattcccagttacattccccactggctaacaatagcacccagtttttcatc
acctttcttcaaatttctcggcgatttgttaaaaacaaaatttgtgtcccttctctgatatctctatgtctctaaac
acaagttcatcggaaaacgaaggagggtaggtgttggttgggctcccgaagtgaaaatagaagagcaagaatagaat
attagagagagagtgcagagagggcgggatagctcccgggattccgttttcttcttctttatcttcaacgatgatgt
gtgtgcgtgttgtatagattctgttgctcccccacaactcgctccgaaggctcaatacaattcaattgatattggag
gagagcctaccggagtgggaggataagaagaaacataagaagaagaagaagaagaagcatgcttctggtttttgatg
ctatgaaaacggcacaaaaagatgattgaggtccctttcaataccttctctcatctttcaaatcccattgaaacct
aaaacttctcaccacgctttaccattgttctccaaaaacttatagcaatgtctataactttttatctctgaaaagc
agtgttccattttctttttcctattttatttcaattgtttctcacatttcgtttggattctttgcttgtcaaccag
cttcttcttccacttttaccgtctaattttcagggcagggagccatcaaacccacgaccactagatccatctagaa

nlg-1 C-terminus

Transmembrane domain indicated in bold. Exons are capitalized, introns are in lowercase.

TTGCTGTCAGATCATTTCCGAAAAGACTCTTACTTTGGAAAAACCCGTCATTTTTCTTCATATGCGAATCTTCCGTT
CCCGCCACCACCAATGCCCCCATCACCACCACCAGAGCTTACAACAAAGCCCAAACCAAGTGAATgtaagttttgat
agttaagttcaaacaaatctagttgtataaacaatacaagttttagagttttagacaagtgagattttgagaaaaa
ataacactgctataaaattcttaattaaatgccttgactcgttttgatagtcgaaaaaaagtcacaaaatactaca
aaacctctagaacccagttttattaagacggatattgaaagcttgttctacaagtttttaacagtgctttcttgtt
ttttccaaatatttctaactctgactggaatttgaaaaaactagttgatattttgaaataacgtttcacgttgaaa
aatatttctagtttcaaacatttttgcccttttgaaataaataactatttcatattcaaacctgccagcttccaccg
gtattaatactccacccgataagttgttttctcatccgaaactagaagaacctaagctgtttgaaaacattattgta
attagaaatgattcagCGCCCACAACTCTCCAAACAACAACTGAAAGTGAAAAGGCCGCCGCTGGAAGTTTTACTGG
AAAAGC**TCTCGGTGGCGTTATTTTCATCGGTTGTGGATTCCTCATTATGAACGTTTGCCTATTAATTGCTGTTCGTA**
GAGAAgtaagttttctgctttgccttcccttcagtagtagttccagtattgttgtcttgaactgtacacgctggtga
tcatgacaatggagtagctatctatcttattcagttgaattttgtcacttgattaagttattttcctgttacacat
tatcgatatttag**TGGGGAAAGAAGCGGCGGAA**CGAGAAGAAGTTTCAACTGCAGTATCAGACTTATAACTCCAACC
ATGGCGGCGGCGCGGAACAATACAACAGCTTAAACTCGCCGgtatgaactatttgcttctcttttgtgaatttgttt
ctagttttttttctcgattgatttgaaccaaaaactaacgaaattcaactagcttaacctttcatccaacgcacgag
catggctcagaactccccctaatccttaatttggttatcagttatccctcatgccaccacctccgccaccgctcaat
ggtgttcacgacgacattttcgatcatcgggtgccgcatcttcggaacggatccacggttcacggcacgcttccgag
gcattcatttcaggaacaggcacgtgtataatgcattatcctctcaccctgtttgaaaatctttttttcaagttatat
ttttctctcgctttttggtatagatttttttccaatttttgaagttgaagtttccaaatggtcgtagaacgaatttgt
tagaaaattgtgaattaataataattaactttcgaaatatggcaattttaacagctcaaaagtaagaaaataatgta

Development • Supplementary information

gtttaaacttaattatgatataacttgccaaatgtaattttgcattttctgactttaaactgataacttattagatt
tttaagcattcattgggcccaatcaaagatttgaaaaattttacagtgtttaaataaatttctcgaaatgagttgac
tcaatttagtagaaaacataatcgattgaaatacaaaacaagcgaatctattctaaaagcactttcctttttttaaa
tacctttcttttaaagttcatatttcaatattcaaataattgcccagttttaaacgagtgtgacattttgaccgttt
cgcacaagttagcagtcacctttcaatttttgcaattcactaacaaaacttgttttgctaccatttgatctaacttc
gaactaaacggaatgacctacctcaacctgcgctttctgatattcataaccttttcattaaaaaataaatgttgcgt
cccttgtccattcagttcttgtaattgtgagatcagtcgaaaacttgtgtgcttttagGAACCCTTACTATCCGCAT
CGCACAAGAACTCAACTTCGATGCGACCCGCGGGAATATCACCAACGTGTCCACGTCACGGACGTGCCGCGCTTGCG
CTTCAAAATAGCCGgtaagtgattgcagcgtgttcttttcatactgacatgttcaagAGGTAACAGTTTGACTGCTG
CTCAAGCACCGACATTGGAAGAGATACAGGTC

Chr IV transgene insertion site (Chr IV:4,237,723)

Guide RNA: ACTGTTGGATGCCTGTGTAG

5’ homology arm (Chr IV:4,236,614 – 4,237,722):
aggaatcttcttcgcggacggaactcgccgctaagcggatcgcctttaatttattctttgccttatgcgttgctcaa
aataaaacattatttcatccaaaataatttttttgaggttttagatgaatttgatagtttagcttgtcactttctcct
gtgcgaagaaacttgctaagcaggtaagcagcatccttatcaagtttcgaccttatctctcagtagtcgcatacgtc
gtatccctcatctgctttgttttagccacttcaagggaattcagatcacttttttctcatgtgttttcaaatttactt
ttgtggagtgaccttaattgaccctctcgtcataagtggacggtgggtagttacttaacaaatttcgagtacaggat
agtgtcaatggcatgagacatgtagtagccaagcaattatggcgaataagtttcaaaaaccaacattctgtcaaata
caaagacctgcaacattagagaattcattaaatacatcttcgaggaaatcaaatttcccgtagacgatcccgtaga
cctacttgtacaatatgttctagatatacactccgaacttaaaatatctcgaacatttgtgttgaatatttattta
attctaggtttttcaaattccgcacgattttgccatactactgcccaatagtttacatttattgggcctggatctg
taaggcacttttgggagctccctttcttttccttcaaattacccatacagatctataaatcacaatttaatacaaga
ttttattcaagttaaaaacataaaaggggctttgcggtggtccccatttcaccagagaactataaaaaatataaatt
taacatcaaatgttgcaattgattccctctcaaagctgcttgaagaaccctgattctgtcaagcctatgaagattta
aaaaaaattgggaagacccttagttccaaacaagtgtcgttgacccagtaggcgatattctgaaaagtcataaaatg
gggttgctaaaaaattggtcgctaacttacatttagctaggaatgttaattggaataactcataatttacagtaaaa
ttatcaaaaaaatagcaaaaatcaatagagga

3’ homology arm (Chr IV 4,237,729 – 4,238,654:
cacaggcatccaacagtacgagttctgcgtttttgaattatagaatcaagcatgctccgcgtttgctgtactatttc
tcgcagagcagttttttttctcacactttattattctgtactgcccctccacgctataatttttttgcatagagggcc
ataaaggaaaacggtacggtaaaaataatgggtcctttttaccgttgtgtagattgtttctaacgggatgcaaattt
tcactaaaactctggaaaactgatggaattctacgggcagccggcaattttagatatttgaagaatccttcttgatc
agtgccaagctttcccatggaccaaagatgtggatccaagatcaaatgcacaggatctgaactcctttgttccaaaa
acaaggatttcttttctatagtttgacctaaatattatagtagaacatatacatttgatgatttcatgaataattta
ttgttaaaaaataatacatgtgttaaaaaataattctaaaaaattaattggagcgtccttggcggatatggcggatt
ggaaggtcataagtaactcgaactaacctgtaaatataattaatttagaagtaatcattttgaaatgtttctaaatt
tttgtaaacgtaatcaatgatttacttacaaaatttttcgaaatattagtagaaatataaaaaaccagtaaaattca
aaagaggccagccacacatgcatcttacaatacgaatgatgcgtttttttgtcgcctcggagaaggaatcaagcaggc
tccgcgtttctgtactatttctcccagagccgtttattttgcataggggtgggtcataaacggagacgttttggt
ataaaatttgccccttcttccaccgttgaaaaaattggaaaatgaaccgggacaggaaaggggatttcaggataatg
gt

Development • Supplementary information

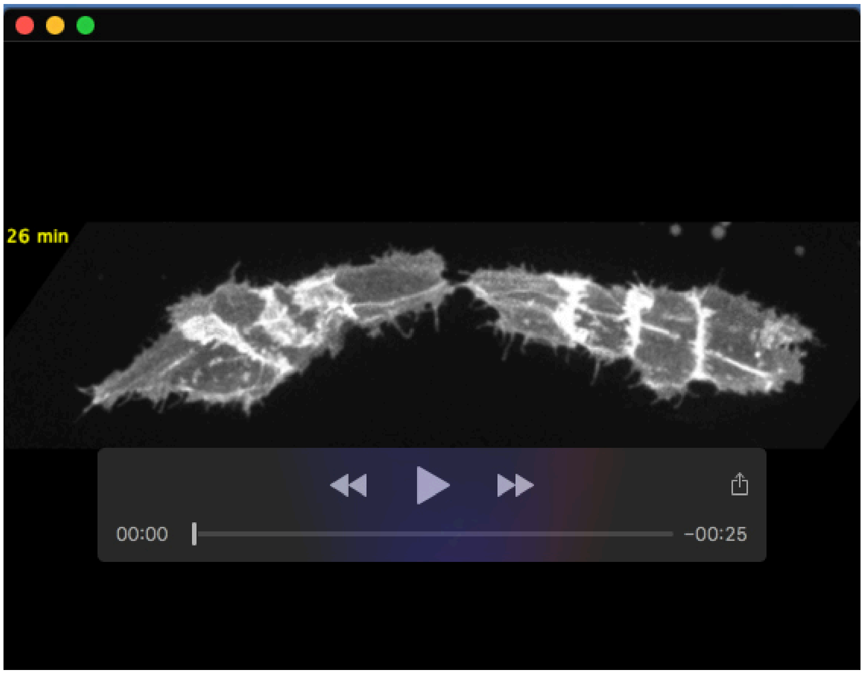

Movie 1. Cytoneme-like protrusions in SMs after migration and proliferation. Spinning disk confocal time-lapse showing cytoneme-like extensions in post-migratory SMs. SM membranes were visualized with the same *Phlh-8>2x mTurquoise2::PH* marker used for imaging SM migration. Movie shows maximum intensity projection of confocal z-slices. Animal is oriented with anterior to left and dorsal to top. Time elapsed is labeled in minutes. Movie at Figshare: https://doi.org/10.6084/m9.figshare.29646680

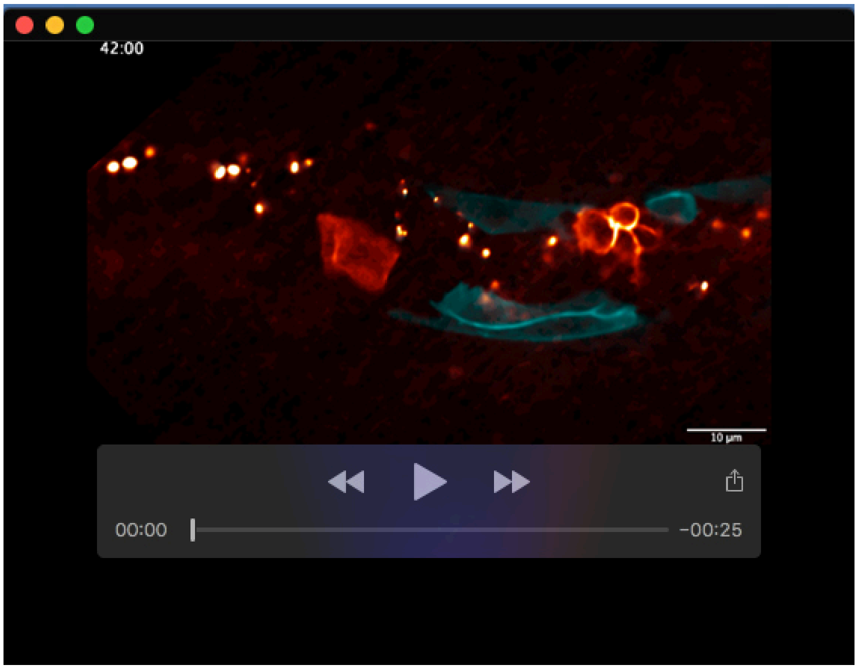

Movie 2. Time-lapse imaging of SM migration in concert with endogenous FGF/egl-17 transcriptional reporter. Spinning disk confocal time-lapse showing a portion of the FGF-dependent phase of SM migration. The SM and other M lineage cells are visualized in cyan. The *FGF/egl-17* transcriptional reported is depicted using the glow lookup table. Movie corresponds to Fig. S5. Animal is oriented with anterior to left and dorsal to top. Time elapsed is labeled in minutes. Scale bar = 10 mm. Figshare: https://doi.org/10.6084/m9.figshare.28611896.v1
